# Role of purinergic receptors in the Alzheimer’s disease

**DOI:** 10.1007/s11302-018-9629-0

**Published:** 2018-10-25

**Authors:** Marek Cieślak, Andrzej Wojtczak

**Affiliations:** 1 Neurology Clinic, Torun, Poland; 2 Department of Crystallochemistry and Biocrystallography, Faculty of Chemistry, Nicolaus Copernicus University in Torun, Gagarina 7, 87-100 Torun, Poland

**Keywords:** Alzheimer’s disease, Purinergic receptors, Adenine nucleotides, Adenosine

## Abstract

Etiology of the Alzheimer’s disease (AD) is not fully understood. Different pathological processes are considered, such as amyloid deposition, tau protein phosphorylation, oxidative stress (OS), metal ion disregulation, or chronic neuroinflammation. Purinergic signaling is involved in all these processes, suggesting the importance of nucleotide receptors (P2X and P2Y) and adenosine receptors (A1, A2A, A2B, A3) present on the CNS cells. Ecto-purines, ecto-pyrimidines, and enzymes participating in their metabolism are present in the inter-cellular spaces. Accumulation of amyloid-β (Aβ) in brain induces the ATP release into the extra-cellular space, which in turn stimulates the P2X7 receptors. Activation of P2X7 results in the increased synthesis and release of many pro-inflammatory mediators such as cytokines and chemokines. Furthermore, activation of P2X7 leads to the decreased activity of α-secretase, while activation of P2Y2 receptor has an opposite effect. Simultaneous inhibition of P2X7 and stimulation of P2Y2 would therefore be the efficient way of the α-secretase activation. Activation of P2Y2 receptors present in neurons, glia cells, and endothelial cells may have a positive neuroprotective effect in AD. The OS may also be counteracted via the purinergic signaling. ADP and its non-hydrolysable analogs activate P2Y13 receptors, leading to the increased activity of heme oxygenase, which has a cytoprotective activity. Adenosine, via A1 and A2A receptors, affects the dopaminergic and glutaminergic signaling, the brain-derived neurotrophic factor (BNDF), and also changes the synaptic plasticity (e.g., causing a prolonged excitation or inhibition) in brain regions responsible for learning and memory. Such activity may be advantageous in the Alzheimer’s disease.

## Introduction

The Alzheimer’s disease (AD) is a progressive and incurable neurodegenerative disease and the most frequent type of dementia in humans older than 65 years. The most important symptom is a progressive cognitive disorder, especially with prominent deficits in short-term memory. This disease was for the first time described in 1907 by German neuropathologist Alois Alzheimer’s and was called by his name [1]. About 20 million people all over the world are affected by AD, what is a serious medical, but also social and economical problem [2]. Majority (circa 85%) of all AD cases is a sporadic form, while 15% is a familial form related to the mutations in the genes of the amyloid precursor protein (APP) and presenilin (PSEN1 and PSEN2). Since age is the major risk factor, the increasing human population and the average life time would increase the number of AD patients. Besides the age, the simultaneous occurrence of genetic causes, the life style, and environmental factors contribute to the development and progress of the disease. Another AD risk factors are the polymorphism of apolipoprotein E (ApoE), or changes in the activity of α-antichymotrypsin (ACT), butyrylcholinesterase (BchE), and oxoglutarate dehydrogenase complex (OGDC) [2].

Many hypotheses have been formulated to explain the pathogenesis of AD. They include different pathological processes such as amyloid deposition, tau protein hyperphosphorylation, metal ion dysregulation, and chronic neuroinflammation processes, which might also be the secondary processes induced as a response to the oxidative stress (OS) [3, 4].

The AD has a characteristic pathomorphological picture. Among the most important pathomorphological symptoms are the extracellular deposition of Aβ which contains 39 to 42 amino acids (as diffuse or focal deposits) in a form of senile amyloid plaques, or intracellular formation of neurofibrilary tangles (NFT). These processes are accompanied by microgliosis, neurite dystrophy, loss of neurons and synapses, and reactive processes including activation of astrocytes and microglia [5–8]. The National Institute on Aging-Alzheimer’s Association has published new recommendations for the postmortem neuropathological assessment of Alzheimer’s disease [8, 9]. The loss of neurons concerns mainly cholinergic neurons, and finally leads to the decrease in the concentration of neurotransmitter acetylcholine in hippocampus and the brain core [7]. Important disturbances in AD are related to changes in the protein structure. Disturbances in the protein folding result in the protein ability to aggregate. In particular, the presence of insoluble amyloid is dangerous for the brain functioning. The presence of amyloid is detected not only in the brain, but also in other organs and is referred as misfolding diseases [10]. The essence of AD is not the formation and aggregation of Aβ, which is resistant to the enzymatic proteolysis, but rather disorders in processes of its elimination, changing the equilibrium between the β-amyloid formation and elimination. The Aβ peptide induces the microglia to generate nitric oxide (NO), reactive oxygen species (ROS), proinflammatory cytokines (IL-1β, IL-6, and IL-18), TNF-α, chemokines, prostaglandins such as PGE_2_, enhances the release of nucleotides that contribute to the cell death [11]. The most important cytokine is IL-1β, since it enhances the expression of other proinflammatory cytokines. The Aβ is released as the amyloid precursor protein (APP). There are two pathways of APP transformation: first with participation of β- and γ-secretases and the second one, important for AD, which involves α-secretases. In the former process, assisted by β- and γ-secretase, the secretion of P11 leads to the formation of β-amyloid. In the non-amyloidogenic process, processing of APP with α-secretase leads to the formation of soluble N-terminal APP protein and P11 peptide called C99 fragment. The long form of β-amyloid (Aβ42) accumulates in the brain forming the insoluble plaques [12]. Currently, this enzyme is an object of the research aiming to design new anti-amyloid drugs [13].

Other characteristic pathomorphological feature of AD is the intracellular aggregation of neurofibrillary tangles (NFT), the major component of which is the hyperphosphorylated tau protein. According to the amyloid cascade hypothesis, the NFTs are formed as a result of toxic action of plaques, and they damage the cellular microtubules, what leads to the disturbances in the inter-cellular transmission. In addition, the synaptic degeneration and the death (usually due to apoptosis, rarely due to necrosis) of selected populations of neuronal cells are observed.

Pathological processes lead to the degeneration of neurons and their axons, and consequently to destruction of the signal transmission pathways and the cell death. Accumulation of Aβ in a form of plaques causes many secondary reactions, including the excessive induction of astroglia and microglia, induction of neuroinflammation, release of cytokines, reactive oxygen species, and nitric oxide [14]. The neuron apoptosis and activation of glia cells lead to the damage of the blood-brain barrier, what facilitates the brain penetration by active immunological cells participating in the spread of inflammatory processes.

Some brain regions are especially susceptible to the pathological changes. In early stages of disease, the pathological changes occur in hippocampus, entorhinal cortex, corpus amygdaloideum, and cerebral cortex [15, 16]. Some reports confirm that the ventricular system with the cerebrospinal fluid participates in the pathogenesis [17]. Several factors are considered as AD markers in the cerebrospinal fluid (CSF), including concentration and ratio of Aβ_42_ to Aβ_43_ and Aβ_40_ to Aβ_42_ peptides, amount of tau protein and the level of its phosphorylation, concentration of apolipoprotein E (ApoE), and probably concentration of dimethylarginines and homocysteine [2, 18]. Purinergic signaling is supposed to participate in that process, since the presence of P2X2, as well as P2X7 and P2Y2 purinoceptors, has been confirmed on the surface of cerebrospinal fluid contacting neurons (CFCN) [19]. Recently, the presence of P2X7 and P2Y2 was detected on the rat choroid plexus of the brain ventricular system [20]. Since the Aβ peptides are present in CSF, and the presence of some purinoceptors is confirmed on the neurons of the ventricular system, we hypothesize that there is a relation between them in the AD etiopathology. Recent research confirmed the role of purinergic signaling in the etiology of many diseases and some new drugs have been developed and implemented in their treatment [21–26]. In particular, drugs affecting the purinergic signaling are used in the treatment of Parkinson’s disease (Istradefylline) or in the ischemic stroke (Ticlopidine, Clopidogrel, Dipyridamole), but might also be used in the treatment of multiple sclerosis, epilepsy, migrene, neuropathic pain, or psychiatric disorders. Reports on the purinergic signaling enlight the possibilities of development and application of such drugs in the treatment of AD, becoming the threat for the aging society. [27]

## Purinergic signaling in the central nervous system

Ecto-purines and ecto-pyrimidines are present in the extracellular milieu of the central nervous system (CNS) and they act as the signaling molecules. These compounds participate in the neurotransmission, induction of inflammatory processes, and modulation of the sensory signals, including conduction of the pain signals. Moreover, nucleotides and nucleosides participate in the blood pressure regulation, in hemostasis with the blood platelet aggregation and in immunological processes.

**Fig. 1:**
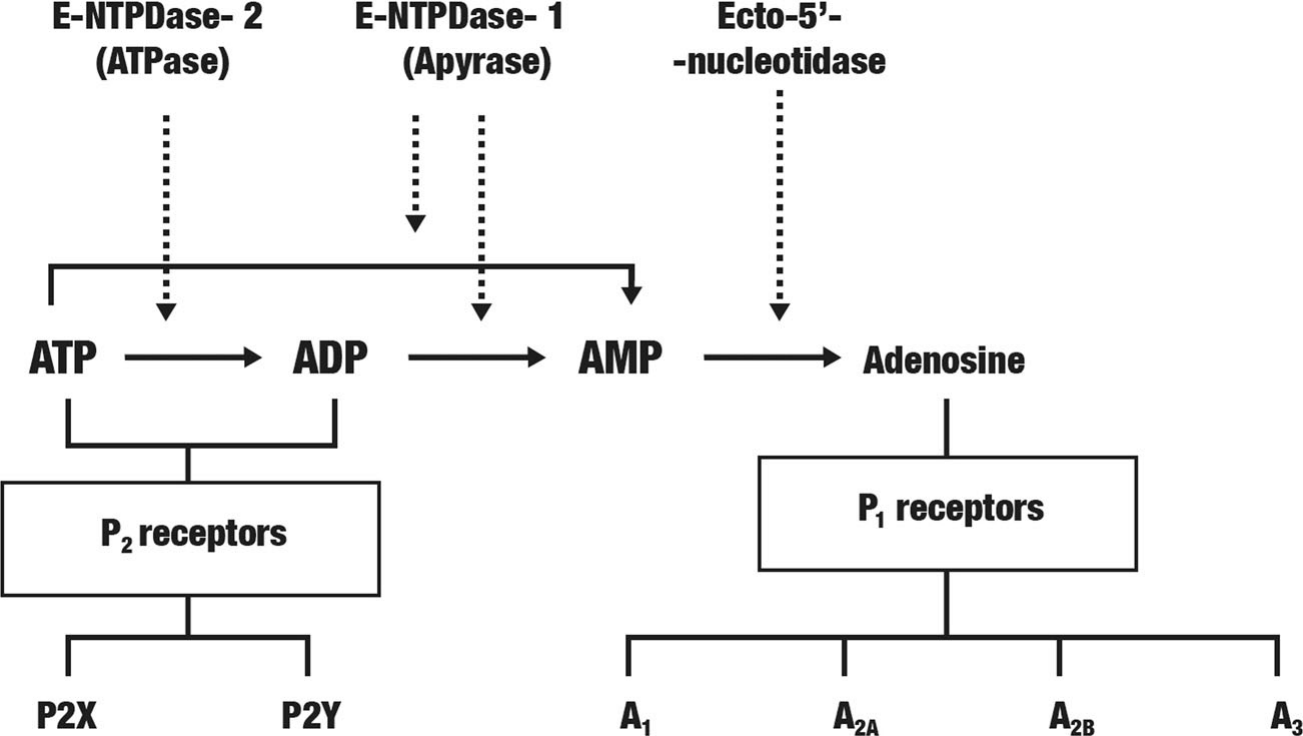
Relationships between ecto-enzymes metabolizing adenosine nucleotides, adenosine nucleotides, and purinergic receptors in CNS, which are related to the etiology of Alzheimer’s disease

In CNS, ATP has a pro-inflammatory and pro-apoptotic action. In turn, adenosine and guanosine are involved in the neuroprotection according to different mechanisms [28]. The complex relations between adenosine nucleotides, the ecto-enzymes metabolizing adenosine nucleotides, and purinergic receptors are shown on Fig. 1. Purinoceptors have been divided into P2 receptors activated by the adenine nucleotides (ATP, ADP) and pyrimidines (UTP and UDP), as well as P1 receptors activated only by adenosine [29]. In turn, P2 receptors are divided into ionotropic P2X and metabotropic P2Y receptors [30, 31]. Both P2X and P2Y, but also nucleoside triphosphate diphosphohydrolases (NTPDases) and 5′-nucleotidase (CD73), are found in all CNS cells - neurons, astrocytes, oligodendrocytes, microglia cells, and on endothelial cells and T lymphocytes. Receptors P2X4, P2X7, P2Y6, and P2Y12 are found on microglia cells [32]. The presence of seven sub-types of P2X and eight sub-types of P2Y in CNS has been reported [33]. Ecto-nucleotidases affect the concentration of ecto-nucleotides, in this way changing the nucleotide-related signaling. ATP and ADP are hydrolyzed by NTPDases and 5′-nucleotidases into adenosine, another signaling molecule stimulating P1 receptors. It was shown that in brain core and hippocampus, the neuron cell membranes reveal high activity of NTPDase1 and NTPDase2. Also, high activity of ecto-5′-nucleotidase and ecto-adenosine deaminase was detected in majority of brain regions while that of adenosine kinase only on astrocytes [34]. The tissue non-specific alkaline phosphatase (TNAP) is a CNS ectoenzyme involved in degradation of ATP to adenosine [35, 36]. It was demonstrated that TNAP activity is significantly increased in the brain in the plasma in both the sporadic and familial forms of AD and that TNAP is related to neuronal toxicity via tau dephosphorylation and could cause the neuronal loss in AD [35].

The G protein-dependent P1 receptors are divided into A1, A2A, A2B, and A3 [37]. In CNS, the adenosine receptors are found on the neurons and glia cells (astrocytes and microglia). Some controversies concern the presence of A2B receptors on the microglia cells. So far, the only reliable report on their presence is that by Koscsó et al., showing that adenosine enhances the production of IL-10 via activation of A2B receptors present on the microglia cells [38]. Adenosine is produced both inside and outside the cells in the process of AMP degradation assisted by 5′-nucleotidases [39]. Also, adenosine can be directly released from neurons and astrocytes via exocytosis. However, the dominating mechanism determining the concentration of adenosine in the extracellular spaces is assisted by bidirectional equilibrative nucleoside transporters (ENTs). The adenosine conversion proceeds by phosphorylation into AMP that is catalyzed by adenosine kinase or by deamination into inosine assisted by adenosine deaminase [39].

Neuroinflammation initiates the neurodegenerative processes, including AD, by activation of adenosine A1-3 receptors, activation of P2X receptors by ATP, and activation of nucleotide P2Y receptors by ATP, ADP, UTP, UDP, and UDP-glucose [6, 40]. However, little is currently known about role of purines in the etiopatogenesis of AD. Possibly, the new insight will be provided by genetic research, such as the recent report by Ansoleaga et al., showing the disorders in the purine metabolism [41]. That research focused on the gene expression in the brain regions such as entorhinal cortex, frontal cortex, or hippocampus-precuneus in the AD Braak and Braak stages I–II, III–IV, and V–VI (Table 1). The AD patients revealed the dysregulations in the messenger RNAs—APRT, DGUOK, POLR3B, ENTPD3, AK5, NME1, NME3, NME5, NME7, and ENTPD2, when compared to the control group [41].

**Table 1:** Braak and Braak stage: this approach evaluates primarily the distribution of neurofibrillary tangles (NFT) within the brain (modified from The National Cell Repository for Alzheimer’s Disease, NCRAD)

Stage	NFT distribution	Typical clinical impression
I/II	Entorhinal	Normal cognition
III/IV	Limbic	Cognitive impairment
V/VI	Neocortical	Alzheimer’s disease

## Relations between the oxidative stress and the cellular levels of nucleotides

The oxidative stress (OS) is a state in which the intracellular generation of reactive oxygen species (ROS) exceeds the efficiency of both non-enzymatic and enzymatic anti-oxidation mechanisms [7]. In AD, the β-amyloid peptide 1-42 (insoluble form) reveals the neurotoxic action via the oxidative stress. Aβ causes the formation of ROS, in particular hydrogen peroxide, which is capable of reacting with the metal ions present in the senile plaques. The oxidative stress results in the damage of proteins, lipids, and nucleic acids. Due to the low efficiency of the antioxidative system, brain is at higher risk related to the OS than other organs. In course of AD, many neurons undergoing apoptosis resulting from the OS reveal high concentration of some proapoptotic proteins such as caspase-3 and Bax (pro-apoptotic protein Bcl-2-associated protein X) [42].

In AD, some significant disturbances occur in the ATP conversion and the neuron energy balance [43]. These disorders affect mitochondria and formation of ATP [44, 45]. It was shown that the reactive oxygen species accumulate in cells, what causes the damage of mitochondria, and that process further enhances the formation of ROS [46]. These processes disturb the function of cells and lead to apoptosis. Damages caused by ROS disturb the electric potential of mitochondria membranes and lead to the increase in concentration of the intracellular ATP [47]. Research by Camandola et al. showed the disorders of Cl^−^-ATPase and Na^+^/K^+^-ATPase in brain of AD patients, what caused the lowering of Na^+^, K^+^, and Cl^−^ ion gradient between cell membranes, leading in consequence to the cellular excitotoxicity and neuronal apoptosis [48, 49]. Therefore, the mitochondria dysfunction in AD consists of lowering the activity of complex IV (cytochrome c oxidase), lowering activity of other enzymes of the tricarboxylic acid cycle, and mutations in mRNA [7]. However, the complete mechanism of complex IV damage is not yet understood.

Research on animals revealed that in the brain cells, especially in astrocytes, the significant increase of ATP concentration leads to the inflammatory processes, resulting in the accumulation of the reactive oxygen species (ROS) [43]. In AD, under oxidative stress, mitochondria do not maintain the level of cell energy by decreased ATP production in neurons [50]. It was also shown that lowering of the energy metabolism by impaired mitochondria, including those damaged under OS, results in occurrence of dysfunctional astrocytes common in AD patients [46, 51]. Finally, the disturbances in the cerebral ATP lead to the impaired cognitive ability. [43]

Currently, the efficient way of mitochondria protection in AD is investigated [47, 52]. Among synthetic antioxidants, the MitoQ, SS31, and ASS234 peptides, as well as donepezil (acetylcholinesterase inhibitor), are investigated [7, 53, 54]. The endogenous compounds of potential antioxidant action include melatonin, glutathione, or peroxiredoxins (Prdx) [7]. The plant-originated compounds of potential antioxidant activity include caffeine, gypenoside XVII (GP-17), and acteoside, an antioxidant phenylethanoid glycoside extracted from *Verbascum sinuatum* named “verbascoside” [7]. The in vivo research on the cellular model of early form of AD showed that the rice bran extract has a protective action against disorders in the mitochondria action [52]. The rice bran extract contains such compounds as oryzanol, vitamin E (tocopherol), and tocotrienols. In that research, use of the rice bran extract resulted in the increase of the cell respiratory index and the intracellular ATP concentration.

The hippocampus samples collected post mortem from patients with AD revealed the high concentration of hemeoxygenase-1 (HO-1), but also, serine phosphorylation was significantly higher than in the control group [55]. The hemeoxygenase-1 and biliverdin reductase-A are considered to protect cells against the oxidative stress. Very few and contradictory reports exist on the effect of the purinergic signaling on the oxidative stress. Research on the animal model showed that ADP and its stable derivatives, such as 2-methyl-thio-ADP (2MeSADP), activate P2Y13 receptor causing an increase in the HO-1 activity in cerebellum neurons and in this way having the cytoprotective action [56]. In contrast, it was shown that ATP and 2MeSADP do not affect the death rate of neurons in the hippocampus region [57]. Research on mice revealed that during the oxidative stress, expression of MCAT (mitochondria-targeted antioxidant catalase) prevents the abnormal APP conversion, reduces Aβ levels, and enhances the activity of Aβ-degrading enzymes [58].

## Role of purinergic signaling of circulatory system in Alzheimer’s disease

Occurrence of AD symptoms is sometimes preceded by pathological changes in the brain vascular system, including accumulation of Aβ in the walls of blood vessels (cerebral amyloid angiopathy) and lowering of cerebral blood flow (CBF) [59]. Research on humans suggests that Aβ causes vasoconstriction of brain vessels triggered by free radical formation [6]. These disorders lead to the brain hypoxia and the damage of the blood-brain barrier. Purinergic signaling participates in both vasoconstriction (vasospasm) and vasodilatation. ATP released from endothelial cells and blood platelets participates in the microcirculation in the brain by activation of P2X and P2Y receptors and the release of the endothelium-derived relaxing factor (EDRF) into the blood. Also, UTP participates in the brain vasodilatation in a process dependent on the endothelial P2Y2 receptors, what results in the lower blood pressure [6]. The released ATP from perivascular sympathetic nerves and damaged endothelial cells can be involved in the mechanism of local vasoconstriction via activation of P2X1 and P2Y2,4,6 receptors present on smooth muscles [25, 60, 61]. Experiments indicate that P2Y1,2,4,6 present on endothelial cells play an important role in preventing the vasoconstriction of brain vessels and lowering the CBF caused by Aβ-triggered release of NO, prostaglandins, and EDHF, and that process seems to be important at the initial stages of AD [6, 61].

## Role of adenosine and adenosine receptors in the Alzheimer’s disease

Adenosine is responsible for the integration and regulation of the neuron activity and affects such physiological processes as sleep and wakefulness, cognitive processes, memory, learning. It also has the neuroprotective action by preventing the neuron damage, what is important in moderating the pathological processes such as neurodegeneration [62, 63]. Since several neurodegenerative diseases may coexist, the common element might be disorders in the adenosine metabolism [8]. In the extracellular space, adenosine affects these processes by activation of P1 receptors (A1, A2A, A2B, and A3) [16]. Activation of P1 also affects the permeability of the blood-brain barrier [64]. Activation of adenosine receptors affects the release of stimulating neurotransmitters (glutamates), what results in either the receptor inhibition (A1, A3) or the increase of their release (A2), affecting the communication between neurons [65]. It is hypothesized that signalization with adenosine, via activation of A1 and A2A, might be a future way of affecting the integrity of dopaminergic and glutaminergic signaling, BNDF (brain-derived neurotrophic factor), and also changing the synaptic plasticity (e.g., provoke the long-lasting excitation or inhibition) in the brain regions responsible for learning and memory [63]. Currently, the A1 and A2A receptor antagonism is the most well-studied potential therapeutic approach involving P1 receptors in neurological disorders. On the other hand, the experimental evidences suggest that A2B and A3 receptors might also be the interesting therapeutic targets in the CNS diseases [66–68].

Research showed that during AD, different activity of adenosine receptors is observed, but also, brain regions significantly differ in their expression. The up-regulation of A1 and A2A receptors in the frontal cortex was detected [69]. In AD patients, the decreased expression and amount of A1 were found in hippocampus, particularly in dentate gyrus, and in CA1 region, while in CA3, the amount of A1 receptors was normal [70–74]. Contrary, the increased amount of A1 and A2A receptors and their increased activity were detected in frontal cortex [69, 72]. Among other brain regions of AD patients, the noticeable decrease in the amount of striatal A1 receptors was reported [75]. Also, the use of positron emission tomography (PET) in AD patients revealed the significantly lower ability of binding [(11)C]MPDX to A1 receptors in temporal and medial temporal cortices and thalamus [76].

Among the brain structures affected by the pathological process, the increased immunoreactivity of A1 receptors was detected in neurofibrillary tangles and on dystrophic neurites of senile plaques. Activation of A1 influences the formation of soluble forms of Aβ and tau protein phosphorylation, what suggests the participation of these receptors in the AD etiology [77]. In this paper, an involvement of A1 in tau phosphorylation by a mechanism involving MAP kinases and its translocation towards cytoskeleton was demonstrated. In the same report, the postmortem examination of AD patients has shown that on neocortical and hippocampal senile plaques, the co-localization of A1 and β-amyloid occurs.

Epidemiological studies in humans have shown the beneficial cognitive effects of caffeine (nonselective A2A receptor antagonist) that have been related to a lower risk of developing AD, but also PD and dementia [78] or reduced age-related deterioration of cognitive abilities [79]. For animal models (transgenic mouse model of AD; AD-like pathology in rabbit hippocampus), the indirect evidences for participation of adenosine A1 and A2A receptors in AD are also reported. That research showed that the non-selective antagonists of A1 and A2A (e.g., caffeine) or coffee consumption have a positive effect for improvement of cognitive functions [63, 80–82]. Results of Cao et al. [80] can be summarized with two conclusions. First, in several mice lines and in aged AD mice, the rapid or long-term administration of caffeine results in lowering the concentrations of Aβ in plasma and brain, suggesting the positive effect of caffeine in AD. Second, the Aβ concentration in plasma does not correlate with the levels of Aβ deposition in the brain, as well as with the cognitive performance in aged AD mice. Giunta et al. have shown that simultaneous inhibition of A1 and A2A protects the neuroblastoma cells exposed to aluminum chloride against the toxic action of β-amyloid [73]. Research on the same cells revealed that activation of A1 results in activation of protein kinase C (PKC), p21 Ras (protein activator 1), and the extracellular signal regulated kinase ERK1/2, what leads to the increase in the amount of the soluble Aβ [72]. These results are very important, since they suggest the A1 involvement in the Aβ metabolism, and also that the use of A1 agonists may be an efficient way of AD treatment even at the late stages.

The in vivo research showed that activation of adenosine A1 and A2A receptors affects the permeability of the blood-brain barrier (BBB) [64]. Their activation enhances the brain penetration by the intravenously administered macromolecules and other molecules, such as dextran and Aβ antibodies. Administration of A1 and A2A agonists in transgenic mice deprived of these receptors resulted in lower penetration of dextran into the brain. These results show that by activating A1 and A2A, adenosine affects the permeability of BBB and in this way facilitates the penetration of macromolecules into the brain. Since the half-life of adenosine is less than 10 s, its role under physiological conditions might be important only at the site of its formation. Drugs with the action mechanism similar to lexiscan or A2A agonists enhancing the permeability of BBB and penetration of molecules into CNS give hope for development of new therapeutic methods for different neurodegenerative disorders including AD.

## Role of adenine nucleotides and P2 receptors in the Alzheimer’s disease

In CNS, the P2 receptors (P2X and P2Y) present on neurons and glia cells (astrocytes, microglia, and oligodendrocytes) affect the degradation of amyloid precursor protein (APP) by metalloproteases, cell differentiation, nociceptive transmission, release of cytokines, and apoptosis [83]. Weisman et al. detected expression of P2Y1,2,4,6,11,12,13,14 receptors in the brain and the presence of P2X3, P2X5, P2X6, P2X7, and P2Y1,2,4,6,12,13 receptors on neurons [33]. Also, in AD patients, the changes in expression of P2Y1,2,4 were reported [6]. Many sub-types of P2 receptors are found on monocytes (P2X1, P2X4, P2X7, and P2Y1,2,4,6,11,12,13) and endothelial cells (P2X4 and P2Y1,2,4,6,11) [84]. In that context, it should be noted that P2 receptors participate in regulation of the cellular immune responses coexisting with the neuroinflammation.

In CNS, ATP is released from cells to the intercellular space according to both lytic and non-lytic mechanism. Activation of P2X7 present on microglia cells is related to generation of superoxide [85]. Recent experiments have proven that in CNS, both ATP and ADP might lower the Aβ concentration in the intercellular space [86]. The tau protein participates in formation of microtubuli cellulares by polymerization of tubulin, which in turn forms a neuron cytoskeleton and plays an important role in the intracellular transport. Disorders associated with an increased production of tau protein are characteristic for the group of neurodegenerative diseases called tauopathies. Neurotoxicity of the tau protein is related to its hyperphosphorylation, conformational changes, and finally aggregation. It was shown that the N-terminal fragment of tau protein contains the ATP and not GTP binding site [87]. Binding of ATP to tau protein induces the tau self-assembly into cellular filaments. Aβ is thought to trigger toxic events, including tau phosphorylation. Accordingly, the depletion of tau prevents Aβ toxicity in AD models.

The generally accepted concept is that in AD, the Aβ induces the phosphorylation of tau protein. It was shown that cognitive dysfunction in AD can be correlated with the tau, but not Aβ pathology [88]. Recently, the research on the mice models of AD [89] proved that at early stages of AD, tau protein phosphorylated at the specific site at postsynapse (T205) has a protective role. These results indicate that tau phosphorylation is involved in normal signaling inter neurons, and it is the aberrant phosphorylation of tau which results in the neuron dysfunction.

Amyloidogenic peptide, oligomeric Aβ42, found in elevated levels of AD patient’s brains, was shown to induce the release of ATP from primary cortical astrocytes in mice [90]. There are many reports on degradation of β-amyloid by IDE protease (insulin-degrading enzyme) [91, 92]. ATP was reported to affect the β-amyloid degradation in a presence of IDE [93, 94]. It was shown that ATP activates the IDE degradation of small peptides, but it decreases the rate degradation of large peptides like Aβ42. The presence of ATP decreases the speed of β-amyloid degradation by factor of 10 [94].

## Receptors P2X

P2X receptor subtypes are widely expressed in the CNS in both neurons and glial cells. They regulate a variety of physiological and pathophysiological processes in the CNS, including neurotransmitter release, neuroinflammation, and pain sensation.

### Receptors P2X7

The P2X7 receptors play a crucial role in the neuroinflammation and neurodegeneration. These receptors, belonging to the family of ionotropic receptors, are activated by ATP in high concentration (> 0.1 mM). P2X7, present on neurons, microglia cells, macrophages, and some lymphocytes, mediates the influx of Ca^2+^ and Na^+^ ions into the cell [95, 96]. Alzheimer’s Aβ peptide was shown to interfere with the P2X7-mediated circadian rhythms of the intracellular calcium levels [97]. Furthermore, recent results [98] show that activation of P2X7 affects alpha-synuclein (ASN)-induced intracellular free calcium mobilization in neuronal cells and leads to the formation of the active complex by pannexin 1, responsible for ATP release. Simultaneously, ASN decreases the ATP degradation by ecto-ATPases. Occurrence of this receptor on the microglia and immunological cells implies its participation in the immunological and neuroinflammatory processes, but also in the neurodegenerative and neuropsychiatric diseases and conduction of neuropathic pain [99]. Activation of P2X7 results in the activation of transcription factors and apoptosis. The especially detriment result of the P2X7 activation is the enhanced release into the intercellular spaces the proinflammatory cytokines, mainly IL1-β, in which elevated levels have been detected in the AD brains, but also IL-18, IL-6, IL1-α, INF-γ, and TNF-α, which are responsible for the progression of Alzheimer’s disease [100]. In the human AD brain, the increased expression of P2X7 receptor has been shown to coexist with activated microglia surrounding Aβ plaques [95]. Moreover, activation of this receptor on the microglia cells by ATP is responsible for generation of hydrogen peroxide [85]. Research of Sanz et al. indicates the negative association between the P2X7R 489C>T polymorphism and AD [101].

It is hypothesized that in AD, β-amyloid in the soluble or fibrillar form induces the neuroinflammatory processes in the brain. Probably, the microglia cells participate in the loss of neurons, in which Aβ is also involved. Research on animal models indicates that Aβ causes the increase in the concentration of intracellular Ca^2+^, release of ATP, and secretion of IL-1β [102].

The current research proved that P2X7 regulates the α-secretase activity according to two opposite mechanisms. It was shown that activation of the P2X7R affects the activity of PKC, MAPKs, or GSK3 [103], which in turn modulate the APP processing and Aβ production. The first mechanism is related to the inhibition of glycogen syntase kinase (GSK-3β). The in vivo research on native J20 hAPP transgenic mouse bearing human APP with K670N/M671L (Swedish) and V717F (Indiana) mutations showed that inhibition of P2X7 on neurons and microglia cells decreased the amount of plaques in hippocampus region [104, 107]. Also, inhibition of native or overexpressed form of P2X7 resulted in the increased activity of α-secretase via inhibition of activity of GSK-3β. The direct inhibition of GSK-3β had the same effect. Research on animals showed that inhibition of P2X7 had a neuroprotective action [105]. Administration of Aβ into the hippocampus region resulted in the increased expression of P2X7, gliosis, increased permeability of BBB, and loss of neurons in the hippocampus region. On the other hand, administration of P2X7 antagonist (brillant blue) decreased the expression of P2X7, gliosis, and BBB permeability and had the neuroprotective action [105].

The second mechanism was reported recently. The research on mouse neuroblastoma cells expressing human APP, human neuroblastoma cells, and mouse astrocytes demonstrated that stimulation of P2X7 activates the enzymatic cascade enhancing the α-secretase activity and leading to the cleavage of APP and formation of soluble and non-neurotoxic sAPPα [106]. In turn, sAPPα formation was inhibited by P2X7 antagonists, knockdown of P2X7 with siRNA or in P2X7-deficient mice. In that research, those modules Erk1/2 and JNK of the mitogen-activated protein kinase are involved in the P2X7R-dependent α-secretase activity. Also, authors showed that knockdown of disintegrin and metalloproteinases ADAM9, ADAM10, and ADAM17 by specific siRNA have not affected the P2X7-dependent non-amyloidogenic processing of APP.

Obtaining the opposite, contradictory effects related to the P2X7 action leading to α-secretase might be explained by the use of different animal models in both investigations and examining of different enzymatic pathways. Possibly, it points out that in AD, both mechanisms of α-secretase activation might be the interesting therapeutic targets.

The intense research was focused on development of β- and γ-secretase inhibitors for the AD treatment. Although different inhibitors have been obtained, their use might be accompanied with the substantial limitations, since downregulation of β- and γ-secretases can cause the significant side effects. Therefore, the alternative way would be the stimulation of α-secretase. That should have the positive effect, since activation of α-secretase was reported to prevent the Aβ deposition and result in a formation of neuroprotective sAPPα [106]. An interesting research showed that stimulation of P2X7 and P2Y2 has the opposite effects on the α-secretase activity. While the P2X7 stimulation decreases its activity, the P2Y2 activation results in its enhancement [107]. Consequently, it might be expected that an increase of the α-secretase activity can be achieved by simultaneous inhibition of P2X7 and stimulation of P2Y2 receptors, and it could be a promising therapeutic targets in AD.

### Other P2X receptors

Participation of neuron P2X3 receptors and P2X2/P2X3 receptor heterotrimers and microglia P2X4 receptors in regulation of pain sensation was reported [68, 108]. Expression of P2X receptor subtypes in the CNS depends on brain region and the cell type. High expression of P2X2, P2X4, and P2X6 receptors was reported for neurons, and their activation was shown to induce both pre- and post-synaptic responses. Although the therapeutic use of P2X7 as potential targets in AD therapy was recently reported [68], other P2X subtypes have not been investigated in that context.

## Receptors P2Y

All subtypes of P2Y receptor present in CNS (P2Y1, P2Y2, P2Y4, P2Y6, P2Y11–14) are activated by ATP, UTP, adenosine 5′-diphosphate (ADP), uridine 5′-diphosphate (UDP), and UDP-glucose [109]. P2Y receptors are expressed in neurons, astrocytes, microglia, and oligodendrocytes. The complex role of P2Y was extensively investigated [6]. In the brain, receptors P2Y affect the neuron activity and function of the neurovascular system, but also participate in the neuroinflammatory processes. Their important beneficial effect is induction of the neuroprotective processes, especially during the neuroinflammation. P2Y affect the conversion of non-amyloidogenic APP, release of cytokines and chemokines, microglia migration, endocytosis of cells by microglia, degradation of neurotoxic Aβ. P2Y receptors also participate in the cellular immune responses to the oxidative stress, axonal outgrowth and neurite extension in neurons, regulation of neurotransmission, and vasodilatation dependent on endothelium [90]. It should be noted that Aβ not only causes the nucleotide release from cells, but also upregulates P2Y, especially at initial stages of AD. In a course of AD, activity of P2Y gradually decreases, as shown by the post-mortem examination/research of human patients.

### Receptors P2Y1

In humans, P2Y1 is wide-spreaded in the brain, especially on neurons of the brain core or hippocampus and on oligodendrocytes [110, 111]. That research showed the presence of P2Y1 on pathological structures which are characteristic for AD, such as neurofibrilary tangles, neuritic plaques, and neuropil threads. Currently, the reason for such distribution and role of P2Y1 in the brain is not known. It is supposed that activation of P2Y1 triggers the neuroprotective action in a response to the increased concentration of extracellular ATP stimulating the receptor and subsequently the release of IL-6 [57]. As reported for animal models [112], the reactive astrogliosis was detected around neuritic plaques. Increase in astrogliosis activity was suppressed by inhibition of P2Y1 receptor, the high expression of which was found on reactive astrocytes present around neuritic plaques. Contrary, the latest results indicate that activation of P2Y1 receptors in astrocytes may contribute to the neurodegeneration [68]. These results suggest that the ability to increase the proliferation of neural stem cells (NSC) through the activation of P2Y1 receptors could be a novel therapeutic way to promote the neuron regeneration in the hippocampus of AD patients.

### Receptors P2Y2

ATP or UTP, by activation of P2Y2 coupled with G_q_ protein, cause the increased activity of phospholipase C (PLC). That leads to the increased production of inositol 1,4,5 trisphosphate (IP_3_) and diacylglycerol, and in consequence to the elevated intracellular concentration of Ca^2+^ and activation of protein kinase C (PKC) [33]. In the brain, P2Y2 is present on neurons, astrocytes, microglia cells of hippocampus and cerebellum regions, and on the endothelial cells [113]. In AD patients, expression of P2Y2 is significantly enhanced on the brain core neurons with the increasing concentration IL-1β [14]. Activation of P2Y2 significantly affects the innate immunity processes such as the monocyte penetration into the damaged tissues, action of neutrophiles in bacterial infections and esinophiles in the pulmonary allergic diseases [114]. The β-amyloid causes the increased ATP release and enhanced expression of P2Y2 on microglia cells and erythrocytes [6]. Such expression of P2Y2 is observed only at initial stages of AD, but it was shown that the P2Y2-assisted neuroprotection gradually diminishes in the course of the disease [114]. Activation of P2Y2 on the brain core neurons causes the increased degradation of APP assisted by α-secretase, what results in a formation of the soluble sAPPα protein rather than the neurotoxic Aβ_1–42_ peptide [6, 115]. In mouse primary microglial cells, activation of receptors P2Y2 by UTP induces the uptake and degradation of Aβ1–42 [116]. In CNS, activation of P2Y2 by ATP and UTP triggers the neuroprotective action related to stimulation and recruitment of microglia cells and neurorepair processes. That proceeds via the Src-dependent transactivation of growth factor receptors, finally leading to ERK1/2 and p38 activation of cell proliferation and neurite outgrowth [90]. For CNS, contradictory effects of P2Y2 activation were reported, depending on the receptor localization, but the predominant opinion is that the activation has neuroprotective effects.

## Possible role of other P2Y receptors in Alzheimer’s disease

Other receptors such as P2Y (P2Y4, P2Y6, P2Y13, and P2Y14) are supposed to be involved in the AD etiopathogenesis, although no strong evidence are reported [6, 117]. Authors emphasize that in AD, stimulation of P2Y4 may trigger the neuroprotective effect and play a significant role in the uptake of Aβ1–42. Similar effects are indicated by experiments revealing in AD the protective role P2Y14 via suppressed expression of metalloprotease-9 [6]. In turn, activation of P2Y13 results in the enhanced expression of transcription factor Nrf2 and the cytoprotective heme-1 oxygenase (HO), what prevents the neuron death in the oxidative stress [56].

## Purinoceptor-mediated response of the central nervous system cells

### Microglia cells

In CNS, microglia cells have important immunoregulation functions, and in different brain disorders, they undergo fast transformation into the active form. The role of microglia in CNS is similar to that of monocytes/macrophages in the peripheral blood [118, 119]. The neuroprotective role of microglia in AD is manifested by their ability to migrate towards the Aβ plaques, neutralization of the neurotoxic activity of Aβ by phagocytosis, and degradation of Aβ [6]. The in vivo research has shown that resident microglia cells migrate fast (1–2 days) towards new plaques [120]. Although there are reports that microglia cells might have either neuroprotective or neurotoxic action [121–123], in the prevailing opinion, their neuroprotective role is dominating. The negative result of activation of microglia cells and astrocytes is release of pro-inflammatory signaling molecules such as cytokines, growth factors, complement molecules, and adhesion molecules [124]. That research has also shown that activated microglia cells release mainly interleukin 1β, while astrocytes release cytokine S100β.

The role of microglia is related to their P2Y2 receptors. Research with the animal model of AD (TgCRND8 mice) showed the increased expression of P2Y2 during initial 10 weeks of life, which diminished after 25 weeks. In the brains of TgCRND8 mice, the P2Y2 receptor is important for activation of microglia cells and might affect the neuroprotective mechanisms via clearance of fibrillar Aβ_1–42_ [114]. P2Y2 receptors present on glia cells affect different intracellular signaling pathways by activating integrins, growth factor receptors, and phospholipase C [90].

In AD, the important role is played by neuroinflammation and activation of immune system. Inflammatory processes are divided into acute (beneficial) and chronic (detrimental). Acute inflammatory processes activate the tissue repair, while the chronic ones lead to the neurodegeneration. In the brain, in the lesion site, the acute inflammatory processes cause the protective effect, since the activated microglia cells release cytokines, chemokines, and most importantly the growth factors participating in the neurorepair [124]. Adenosine participates in these processes by stimulation of cell proliferation and angiogenesis [125, 126]. Both mechanisms and the timescale of transformation of the acute neuroinflammation into chronic one are still unknown. In the initial stage of the chronic neuroinflammation, the microglia cells are activated, subsequently releasing ATP and cytokines into the extracellular space [124]. In turn, the released ATP stimulates the P2Y2 receptors on microglia, what leads to further release of cytokines and spreading of the inflammatory process. The prolonged release of pro-inflammatory factors (e.g., IL-1β) causes the enhanced expression of P2Y2 on neurons and glia cells, while IL-1β, IL-6, and TNF-α cause the intensified microglia migration and proliferation [33]. Moreover, P2Y2 agonists ATP and UTP, released from apoptotic cells, induce the migration of cells capable of phagocytosis [127]. According to the amyloid cascade/neuroinflammation hypothesis, deposition of Aβ results in prolonged microglia activation with detrimental neuroinflammatory reaction caused by the release of pro-inflammatory cytokines, neurotoxins, and free radicals that cause the neurodegeneration [128, 129].

The above results indicate that in designing new way of AD treatment, one has to take into consideration the interruption of transformation of the acute neuroinflammatory process into the chronic process, what should be linked to the decreased reactivity of P2Y2 receptor (e.g., by the use of its antagonist).

### Astrocytes

In AD, the reactive astrocytes are frequently found near plaques, and astrogliosis intensifies with the progression of pathological changes. Aβ plays an important role in their activation. Currently, the AD research is focused on the role of reactive astrocytes in the degradation of extracellular Aβ deposits and in immunological processes [8]. One of the roles of astrocytes is preventing the penetration of the brain/blood barrier by noxiousness substances that might participate in the neurodegeneration [130]. Astrocytes release the neurotrophic factors involved in the neuronal survival [131]. The most important cytokine released by the activated astrocytes is S100β, the overexpression of which was found in the brain of the AD patients [132]. Interaction of P2Y2 with integrins activates the astrocyte migration. Regulation of synaptic transmission is related to the interactions between astrocytes and neurons [133]. It is currently known that formation of the gap functions between adjacent astrocytes involves connexin 30 (Cx30) and connexin 43 (Cx43) [131]. However, the precise mechanism of astrocyte involvement in the physiology of synaptic transmission is still not known.

During the neuroinflammation, astrocytes undergo the morphological and functional transformation, what is referred as the reactive gliosis. During that transformation, the hypertrophy and proliferation occur. The oligomeric β-amyloid peptide Aβ_1–42_ causes the increased release of nucleotides from astrocytes, what results in the stimulation of the neuron P2Y2 receptors. In the brains of AD patients, the reactive astrogliosis is not only accompanied by activation of migration and proliferation of glia cells, but also activation of phagocytosis of cells damaged during apoptosis. By activation of G_q_-dependent receptor P2Y2 on glia cells, ATP and UDP affect different metabolic processes, but also the cell migration and proliferation, which are associated with the reactive astrogliosis. Research on rats showed the upregulation of the astrocyte P2Y2 in the brain core and nucleus accumbens as a result of the mechanical injury [134]. Supposedly, similar mechanism occurs during neurodegeneration.

### Neurons

Activation of the microglia P2X7 enhances the release of ATP, IL-1β, and INF-α. In turn, ATP released from microglia activates P2Y2 receptor on neurons and other cells. In addition, activation of NF-κB (nuclear factor kappa-light-chain-enhancer of activated B cells) in neuroinflammation triggers the IL-1β release, what results in the upregulation of P2Y2 receptors on neurons [115]. That process enhances the nucleoside-induced non-amyloidogenic processing of APP, cofilin phosphorylation, and the neuroprotective processes [90]. Increased activity of the neuron P2Y2 caused by pro-inflammatory cytokines assists the neuroprotective responses, including activation of the non-amyloidogenic APP processing [115]. Therefore, in AD, the increased activity of P2Y2 and agonists of that receptor might have the neuroprotective role related to the inhibition of neuroinflammatory processes.

### Endothelial cells

Activation of P2Y1,2,4,6 receptors present on endothelial cells results in vasodilation via the release of nitric oxide (NO), endothelium-derived hyperpolarizing factor (EDHF), and prostaglandins [61]. Therefore, stimulation of these receptors might prevent the vasoconstrictive action of Aβ. Activation of P2Y2 on endothelial cells causes binding of monocytes to the endothelial wall and their diapedesis, and that process enhances the neuroprotective action of the microglia cells [33].

## Summary

The research summarized here points out to several ways of AD treatment targeting the purinoceptors. Aβ accumulating in the brain induces the release of ATP into the extracellular milieu, which in turn activates P2X7 receptors and leads to the enhanced generation and release of many pro-inflammatory factors. The P2X7 antagonists might diminish the intensity of neuroinflammation and decrease the amount of amyloid plaques in hippocampus. Currently, potential anti-AD drugs are investigated, including inhibitors of γ-secretase, inhibitors of Aβ aggregation, anti-amyloid factors such as neprilysin, insulin-degrading enzyme IDE, and Aβ antibodies. Also, activation of α-secretase prevents the β-amyloid deposition and causes the formation of the neuroprotective fragment sAPPα. Activation of P2X7 receptor leads to the decreased activity of α-secretase, while stimulation of P2Y2 has the opposite effect. Therefore, an interesting perspective for AD treatment based on the α-secretase activation would be the simultaneous suppression of P2X7 and stimulation of P2Y2.

Oxidative stress is one of the factors responsible for the disorders related to AD. Purinergic signaling might be the possible way of counteracting the oxidative stress in AD. The potential anti-oxidative compounds are ADP and its stable derivatives, which activate P2Y13. Stimulation of this receptor results in the increased activity of heme oxygenase, which has the cytoprotective action. Another research direction is a search for the ways of mitochondria protection. Such action is reported for donepezil and many other synthetic and endogenous compounds, as well as plant-derived compounds (e.g., rice bran extract).

Chronic neuroinflammation leads to the neurodegeneration. Contrary, the acute inflammatory processes have a beneficial effect by supporting the Aβ clearance, since they trigger the tissue repair and limit the area of the brain damage. Therefore, disruption of transformation of acute neuroinflammation into the chronic process should be considered as a way of AD treatment. In AD, the activated glia cells play an important role in the inflammation processes due to their ability for endocytosis and consequently the Aβ degradation. The neuroprotective role of microglia in AD is manifested by its ability to migrate towards the Aβ plaques, neutralization of Aβ neurotoxicity, as well as phagocytosis and degradation of Aβ.

Increased concentration of extracellular nucleotides released by activated glia or apoptotic cells during inflammatory processes is reported at early stages of AD. Activation of P2Y2 by ATP and UTP triggers the neuroprotective processes (i.e., enhanced axonal elongation/neurite outgrowth). Activation of P2Y2 on migrating microglia cells also affects the uptake of fibrillar fAβ_1–42_ and oAβ_1–42_ (fibrillar Aβ_1–42_ and oligomeric Aβ_1–42_). However, the significant neuroprotective role of P2Y2 occurs only in the initial phase of AD, when the expression of P2Y2 is enhanced. Also, some reports indicate that activation of P2Y1 on astrocytes might contribute to neurodegeneration, although in neural stem cells, it seems to enhance the neurogenesis.

The purinergic signaling in the vascular system is supposed to play an important role at early stages of AD. Aβ causes the vasoconstriction and decrease of the regional cerebral blood flow, but also accumulates in walls of the blood vessels (cerebral amyloid angiopathy). Therefore, another therapeutic way would be the activation of endothelial P2Y, in particular P2Y1,2,4,6, that might counteract the vasoconstriction of brain vessels via the release of NO, prostaglandins, and EDHF.

Neuronal A1 and A2A adenosine receptors have primarily been targeted to promote the processing of non-amyloidogenic amyloid precursor protein (APP) and prevent the Aβ-induced neurotoxicity. Therefore, moderation of the adenosine-related signalization involving A1 and A2A receptors might be used to affect the dopaminergic and glutaminergic signaling, release of the brain-derived neurotrophic factor (BNDF), what affects the synaptic plasticity (e.g., causing the long-term stimulation or suppression). Activation of A1 by adenosine or its analogs causes the activation of protein kinase C, p21 Ras (protein activator 1), and extracellular signal-regulated kinase ERK1/2, leading to the increase in the amount of soluble β-amyloid. Since A1 and A2A adenosine receptors seem to be involved in Aβ metabolism, at the advanced stages of AD, the A1 agonists and A2A antagonists might provide the efficient way for AD treatment as novel drug targets.

## References

[1] Alzheimer A (1907) Über eine eigenartige Erkrankungen der Hirnride (on a peculiar disease of the cerebral cortex). Allgemeine Zeitschrift fur Psychiatrie und Psychisch-Gerichtliche Medizin 64:146–148

[2] Kubis A, Janusz M (2008). Choroba Alzheimera – nowe możliwości terapeutyczne oraz stosowane modele eksperymentalne. Postępy Hig Med Dosw.

[3] Webber KM, Raina AK, Marlatt MW, Zhu X, Prat MI, Morelli L, Casadesus G, Perry G, Smith MA (2005). The cell cycle in Alzheimer disease: a unique target for neuropharmacology. Mech Ageing Dev.

[4] Niranjan R (2013). Molecular basis of etiological implications in Alzheimer's disease: focus on neuroinflammation. Mol Neurobiol.

[5] Selko DJ (1991). The molecular pathology of Alzheimer’s disease. Neuron.

[6] Erb L, Cao C, Ajit D, Weisman GA (2015). P2Y receptors in Alzheimer's disease. Biol Cell.

[7] Fernández-Moriano C, González-Burgos E, Gómez-Serranillos MP (2015). Mitochondria-targeted protective compounds in Parkinson's and Alzheimer's diseases. Oxidative Med Cell Longev.

[8] Boison D, Aronica E (2015). Comorbidities in neurology: is adenosine the common link?. Neuropharmacology.

[9] Kovacs GG, Gelpi E (2012). Clinical neuropathology practice news 3-2012: the "ABC" in AD-revised and updated guideline for the neuropathologic assessment of Alzheimer's disease. Clin Neuropathol.

[10] Anfinsen CB (1973). Principles that goven the folding of protein chains. Science.

[11] Akiyama H, Barger S, Barnum S, Bradt B, Bauer J, Cole GM, Cooper NR, Eikelenboom P, Emmerling M, Fiebich BL, Finch CE, Frautschy S, Griffin WS, Hampel H, Hull M, Landreth G, Lue L, Mrak R, Mackenzie IR, McGeer PL, O'Banion MK, Pachter J, Pasinetti G, Plata-Salaman C, Rogers J, Rydel R, Shen Y, Streit W, Strohmeyer R, Tooyoma I, Van Muiswinkel FL, Veerhuis R, Walker D, Webster S, Wegrzyniak B, Wenk G, Wyss-Coray T (2000). Inflammation and Alzheimer's disease. Neurobiol Aging.

[12] Jarrett JT, Berger EP, Lansbury PT (1993). The carboxy terminus of the beta amyloid protein is critical for the seeding of amyloid formation: implications for the pathogenesis of Alzheimer's disease. Biochemistry.

[13] Citron M (2010). Alzheimer's disease: strategies for disease modification. Nat Rev Drug Discov.

[14] Lee YJ, Han SB, Nam SY, Oh KW, Hong JT (2010). Inflammation and Alzheimer's disease. Arch Pharm Res.

[15] Kumar P, Clark M (2005). Degenerative neuronal diseases. Kumar and Clark Clinical Medicine.

[16] Rahman A (2009). The role of adenosine in Alzheimer's disease. Curr Neuropharmacol.

[17] Zetterberg H, Lautner R, Skillbäck T, Rosén C, Shahim P, Mattsson N, Blennow K (2014). CSF in Alzheimer's disease. Adv Clin Chem.

[18] Arlt S, Schwedhelm E, Kölsch H, Jahn H, Linnebank M, Smulders Y, Jessen F, Böger RH, Popp J (2012). Dimethylarginines, homocysteine metabolism, and cerebrospinal fluid markers for Alzheimer's disease. J Alzheimers Dis.

[19] Stoeckel ME, Uhl-Bronner S, Hugel S (2003). Cerebrospinal fluid contacting neurons in the rat spinal cord, a γ-aminobutiric acidergic system expressing the P2X2 subunit of purynergic receptors, PSA-NCAM, and GAP-43 immuoreactivities: light and electron microscopic study. J Comp Neurol.

[20] Czarnecka J, Roszek K, Jabłoński A, Smoliński DJ, Komoszyński M (2011). Some aspects of purinergic signaling in the ventricular system of porcine brain. Acta Vet Scand.

[21] Cieślak M, Kukulski F, Komoszyński M (2011). Emerging role of extracellular nucleotides and adenosine in multiple sclerosis. Purinergic Signal.

[22] Cieślak M, Komoszyński M, Wojtczak A (2008). Adenosine a(2A) receptors in Parkinson's disease treatment. Purinergic Signal.

[23] Cieślak M, Wojtczak A, Komoszyński M (2017). Role of the purinergic signaling in epilepsy. Pharmacol Rep.

[24] Cieślak M, Czarnecka J, Roszek K (2016). The roles of purinergic signaling in psychiatric disorders. Acta Biochim Pol.

[25] Cieślak M, Czarnecka J, Roszek K, Komoszyński M (2015). The role of purinergic signaling in the etiology of migraine and novel antimigraine treatment. Purinergic Signal.

[26] Cieślak M, Wojtczak A, Cieślak M (2013). Relationship between the induction of inflammatory processes and infectious diseases in patients with ischemic stroke. Acta Biochim Pol.

[27] Burnstock G (2017). Purinergic Signalling: therapeutic developments. Front Pharmacol.

[28] Wardas J (2002). Neuroprotective role of adenosine in the CNS. Pol J Pharmacol.

[29] Burnstock G, Straub RW, Bolis L (1978). Cell Membrane Receptors for Drugs and Hormones: A Multidisciplinary Approach.

[30] Burnstock G, Kennedy C (1985). Is there a basis for distinguishing two types of P2-purinoceptor?. Gen Pharmacol.

[31] Ralevic V, Burnstock G (1998). Receptors for purines and pyrimidines. Pharmacol Rev.

[32] Di Virgilio F, Ceruti S, Bramanti P, Abbracchio MP (2009). Purinergic signalling in inflammation of the central nervous system. Trends Neurosci.

[33] Weisman GA, Ajit D, Garrad R, Peterson TS, Woods LT, Thebeau C, Camden JM, Erb L (2012). Neuroprotective roles of the P2Y(2) receptor. Purinergic Signal.

[34] Kukulski F, Sevigny J, Komoszyński M (2004). Comparative hydrolysis of extracellular adenine nucleotides and adenosine in synaptic membranes from porcine brain cortex, hippocampus, cerebellum and medulla oblongata. Brain Res.

[35] Kellett KA, Hooper NM (2015). The role of tissue non-specific alkaline phosphatase (TNAP) in neurodegenerative diseases: Alzheimer's disease in the focus. Subcell Biochem.

[36] Sebastián-Serrano Á, de Diego-García L, Martínez-Frailes C, Ávila J, Zimmermann H, Millán JL, Miras-Portugal MT, Díaz-Hernández M (2014). Tissue-nonspecific alkaline phosphatase regulates purinergic transmission in the central nervous system during development and disease. Comput Struct Biotechnol J.

[37] Fredholm BB, Chen JF, Cunha RA, Svenningsson P, Vaugeois JM (2005). Adenosine and brain function. Int Rev Neurobiol.

[38] Koscsó B, Csóka B, Selmeczy Z, Himer L, Pacher P, Virág L, Haskó G (2012). Adenosine augments IL-10 production by microglial cells through an A2B adenosine receptor-mediated process. J Immunol.

[39] Wardas J (2008). Potential role of adenosine A2A receptors in the treatment of schizophrenia. Front Biosci.

[40] Burnstock G (2016). P2X ion channel receptors and inflammation. Purinergic Signal.

[41] Ansoleaga B, Jové M, Schlüter A, Garcia-Esparcia P, Moreno J, Pujol A, Pamplona R, Portero-Otín M, Ferrer I (2015). Deregulation of purine metabolism in Alzheimer's disease. Neurobiol Aging.

[42] Eckert A, Keil U, Marques CA, Bonert A, Frey C, Schüssel K, Müller WE (2003). Mitochondrial dysfunction, apoptotic cell death, and Alzheimer's disease. Biochem Pharmacol.

[43] Thomas SC, Alhasawi A, Appanna VP, Auger C, Appanna VD (2015). Brain metabolism and Alzheimer's disease: the prospect of a metabolite-based therapy. J Nutr Health Aging.

[44] Ferreira IL, Resende R, Ferreiro E, Rego AC, Pereira CF (2010). Multiple defects in energy metabolism in Alzheimer's disease. Curr Drug Targets.

[45] Marazziti D, Baroni S, Picchetti M, Landi P, Silvestri S, Vatteroni E, Catena Dell'Osso M (2012). Psychiatric disorders and mitochondrial dysfunctions. Eur Rev Med Pharmacol Sci.

[46] Castellani R, Hirai K, Aliev G, Drew KL, Nunomura A, Takeda A, Cash AD, Obrenovich ME, Perry G, Smith MA (2002). Role of mitochondrial dysfunction in Alzheimer's disease. J Neurosci Res.

[47] Procaccio V, Bris C, Chao de la Barca JM, Oca F, Chevrollier A, Amati-Bonneau P, Bonneau D, Reynier P (2014). Perspectives of drug-based neuroprotection targeting mitochondria. Rev Neurol (Paris).

[48] Camandola S, Mattson MP (2011). Aberrant subcellular neuronal calcium regulation in aging and Alzheimer's disease. Biochim Biophys Acta.

[49] de Lores Arnaiz GR, Ordieres MG (2014). Brain Na(+), K(+)-ATPase activity in aging and disease. Int J Biomed Sci.

[50] Zhang C, Rissman RA, Feng J (2015). Characterization of ATP alternations in an Alzheimer's disease transgenic mouse model. J Alzheimers Dis.

[51] Wang X, Wang W, Li L, Perry G, Lee HG, Zhu X (2014). Oxidative stress and mitochondrial dysfunction in Alzheimer's disease. Biochim Biophys Acta.

[52] Hagl S, Grewal R, Ciobanu I, Helal A, Khayyal MT, Muller WE, Eckert GP (2015). Rice bran extract compensates mitochondrial dysfunction in a cellular model of early Alzheimer's disease. J Alzheimers Dis.

[53] McManus MJ, Murphy MP, Franklin JL (2011). The mitochondria-targeted antioxidant MitoQ prevents loss of spatial memory retention and early neuropathology in a transgenic mouse model of Alzheimer's disease. J Neurosci.

[54] Ye CY, Lei Y, Tang XC, Zhang HY (2015). Donepezil attenuates Aβ-associated mitochondrial dysfunction and reduces mitochondrial Aβ accumulation in vivo and in vitro. Neuropharmacology.

[55] Barone E, Di Domenico F, Sultana R, Coccia R, Mancuso C, Perluigi M, Butterfield DA (2012). Heme oxygenase-1 posttranslational modifications in the brain of subjects with Alzheimer disease and mild cognitive impairment. Free Radic Biol Med.

[56] Espada S, Ortega F, Molina-Jijón E, Rojo AI, Pérez-Sen R, Pedraza-Chaverri J, Miras-Portugal MT, Cuadrado A (2010). The purinergic P2Y(13) receptor activates the Nrf2/HO-1 axis and protects against oxidative stress-induced neuronal death. Free Radic Biol Med.

[57] Fujita T, Tozaki-Saitoh H, Inoue K (2009). P2Y1 receptor signaling enhances neuroprotection by astrocytes against oxidative stress via IL-6 release in hippocampal cultures. Glia.

[58] Mao P, Manczak M, Calkins MJ, Truong Q, Reddy TP, Reddy AP, Shirendeb U, Lo HH, Rabinovitch PS, Reddy PH (2012). Mitochondria-targeted catalase reduces abnormal APP processing, amyloid β production and BACE1 in a mouse model of Alzheimer's disease: implications for neuroprotection and lifespan extension. Hum Mol Genet.

[59] Sagare AP, Bell RD, Zlokovic BV (2013). Neurovascular dysfunction and faulty amyloid β-peptide clearance in Alzheimer disease. J Alzheimers Dis.

[60] Burnstock G (1989). The role of adenosine triphosphate in migraine. Biomed Pharmacother.

[61] Burnstock G, Ralevic V (2014). Purinergic signaling and blood vessels in health and disease. Pharmacol Rev.

[62] Takahashi RN, Pamplona FA, Prediger RD (2008). Adenosine receptor antagonists for cognitive dysfunction: a review of animal studies. Front Biosci.

[63] Chen JF (2014). Adenosine receptor control of cognition in normal and disease. Int Rev Neurobiol.

[64] Carman AJ, Mills JH, Krenz A, Kim DG, Bynoe MS (2011). Adenosine receptor signaling modulates permeability of the blood-brain barrier. J Neurosci.

[65] Ribeiro JA, Sebastião AM, de Mendonça A (2002). Adenosine receptors in the nervous system: pathophysiological implications. Prog Neurobiol.

[66] Popoli P, Pepponi R (2012). Potential therapeutic relevance of adenosine A2B and A2A receptors in the central nervous system. CNS Neurol Disord Drug Targets.

[67] Little JW, Ford A, Symons-Liguori AM, Chen Z, Janes K, Doyle T, Xie J, Luongo L, Tosh DK, Maione S, Bannister K, Dickenson AH, Vanderah TW, Porreca F, Jacobson KA, Salvemini D (2015). Endogenous adenosine A3 receptor activation selectively alleviates persistent pain states. Brain.

[68] Woods LT, Ajit D, Camden JM, Erb L, Weisman GA (2016). Purinergic receptors as potential therapeutic targets in Alzheimer's disease. Neuropharmacology.

[69] Albasanz JL, Perez S, Barrachina M, Ferrer I, Martín M (2008). Up-regulation of adenosine receptors in the frontal cortex in Alzheimer's disease. Brain Pathol.

[70] Jansen KL, Faull RL, Dragunow M, Synek BL (1990). Alzheimer's disease: changes in hippocampal N-methyl-D-aspartate, quisqualate, neurotensin, adenosine, benzodiazepine, serotonin and opioid receptors--an autoradiographic study. Neuroscience.

[71] Kalaria RN, Sromek S, Wilcox BJ, Unnerstall JR (1990). Hippocampal adenosine A1 receptors are decreased in Alzheimer's disease. Neurosci Lett.

[72] Burnstock G, Krügel U, Abbracchio MP, Illes P (2011). Purinergic signalling: from normal behaviour to pathological brain function. Prog Neurobiol.

[73] Giunta S, Andriolo V, Castorina A (2014). Dual blockade of the A1 and A2A adenosine receptor prevents amyloid beta toxicity in neuroblastoma cells exposed to aluminum chloride. Int J Biochem Cell Biol.

[74] Gaisler-Salomon I, Kravitz E, Feiler Y, Safran M, Biegon A, Amariglio N, Rechavi G (2014). Hippocampus-specific deficiency in RNA editing of GluA2 in Alzheimer's disease. Neurobiol Aging.

[75] Stone TW, Ceruti S, Abbracchio MP (2009). Adenosine receptors and neurological disease: neuroprotection and neurodegeneration. Handb Exp Pharmacol.

[76] Fukumitsu N, Ishii K, Kimura Y, Oda K, Hashimoto M, Suzuki M, Ishiwata K (2008). Adenosine a(1) receptors using 8-dicyclopropylmethyl-1-[(11)C]methyl-3-propylxanthine PET in Alzheimer's disease. Ann Nucl Med.

[77] Angulo E, Casadó V, Mallol J, Canela EI, Viñals F, Ferrer I, Lluis C, Franco R (2003). A1 adenosine receptors accumulate in neurodegenerative structures in Alzheimer disease and mediate both amyloid precursor protein processing and tau phosphorylation and translocation. Brain Pathol.

[78] Eskelinen MH, Kivipelto M (2010). Caffeine as a protective factor in dementia and Alzheimer's disease. J Alzheimers Dis.

[79] van Gelder BM, Buijsse B, Tijhuis M, Kalmijn S, Giampaoli S, Nissinen A, Kromhout D (2007). Coffee consumption is inversely associated with cognitive decline in elderly European men: the FINE study. Eur J Clin Nutr.

[80] Cao C, Cirrito JR, Lin X, Wang L, Verges DK, Dickson A, Mamcarz M, Zhang C, Mori T, Arendash GW, Holtzman DM, Potter H (2009). Caffeine suppresses amyloid-beta levels in plasma and brain of Alzheimer's disease transgenic mice. J Alzheimers Dis.

[81] Arendash GW, Cao C (2010). Caffeine and coffee as therapeutics against Alzheimer's disease. J Alzheimers Dis.

[82] Prasanthi JR, Dasari B, Marwarha G, Larson T, Chen X, Geiger JD, Ghribi O (2010). Caffeine protects against oxidative stress and Alzheimer's disease-like pathology in rabbit hippocampus induced by cholesterol-enriched diet. Free Radic Biol Med.

[83] Burnstock G, Verkhratsky A (2010). Long-term (trophic) purinergic signalling: purinoceptors control cell proliferation, differentiation and death. Cell Death Dis.

[84] Wang L, Karlsson L, Moses S, Hultgårdh-Nilsson A, Andersson M, Borna C, Gudbjartsson T, Jern S, Erlinge D (2002). P2 receptor expression profiles in human vascular smooth muscle and endothelial cells. J Cardiovasc Pharmacol.

[85] Parvathenani LK, Tertyshnikova S, Greco CR, Roberts SB, Robertson B, Posmantur R (2003). P2X7 mediates superoxide production in primary microglia and is up-regulated in a transgenic mouse model of Alzheimer's disease. J Biol Chem.

[86] Coskuner O, Murray IVJ (2014). Adenosine triphosphate (ATP) reduces amyloid-β protein misfolding in vitro. J Alzheimers Dis.

[87] Farid M, Corbo CP, Alonso Adel C (2014). Tau binds ATP and induces its aggregation. Microsc Res Tech.

[88] Serrano-Pozo A, Frosch MP, Masliah E, Hyman BT (2011). Neuropathological alterations in Alzheimer disease. Cold Spring Harb Perspect Med.

[89] Ittner A, Chua SW, Bertz J, Volkerling A, van der Hoven J, Gladbach A, Przybyla M, Bi M, van Hummel A, Stevens CH, Ippati S, Suh LS, Macmillan A, Sutherland G, Kril JJ, Silva AP, Mackay JP, Poljak A, Delerue F, Ke YD, Ittner LM (2016). Site-specific phosphorylation of tau inhibits amyloid-b toxicity in Alzheimer’s mice. Science.

[90] Peterson TS, Camden JM, Wang Y, Seye CI, Wood WG, Sun GY, Erb L, Petris MJ, Weisman GA (2010). P2Y2 nucleotide receptor-mediated responses in brain cells. Mol Neurobiol.

[91] McDermott JR, Gibson AM (1997). Degradation of Alzheimer's beta-amyloid protein by human and rat brain peptidases: involvement of insulin-degrading enzyme. Neurochem Res.

[92] Haque R, Nazir A (2014). Insulin-degrading enzyme: a link between Alzheimer's and type 2 diabetes mellitus. CNS Neurol Disord Drug Targets.

[93] da Cruz CH, Seabra G (2014). Molecular dynamics simulations reveal a novel mechanism for ATP inhibition of insulin degrading enzyme. J Chem Inf Model.

[94] da Cruz CH, Seabra GM (2015). QM/MM simulations of amyloid-β 42 degradation by IDE in the presence and absence of ATP. J Chem Inf Model.

[95] McLarnon JG, Ryu JK, Walker DG, Choi HB (2006). Upregulated expression of purinergic P2X(7) receptor in Alzheimer disease and amyloid-beta peptide-treated microglia and in peptide-injected rat hippocampus. J Neuropathol Exp Neurol.

[96] Volonte C, Apolloni S, Skaper SD, Burnstock G (2012). P2X7 receptors: channels, pores and more. CNS Neurol Disord Drug Targets.

[97] Wilkaniec A, Schmitt K, Grimm A, Strosznajder JB, Eckert A (2016). Alzheimer's amyloid-β peptide disturbs P2X7 receptor-mediated circadian oscillations of intracellular calcium. Folia Neuropathol.

[98] Wilkaniec A, Gąssowska M, Czapski GA, Cieślik M, Sulkowsk G, Adamczyk A (2017). P2X7 receptor-pannexin 1 interaction mediates extracellular alpha-synuclein-induced ATP release in neuroblastoma SH-SY5Y cells. Purinergic Signal.

[99] Takenouchi T, Sekiyama K, Sekigawa A, Fujita M, Waragai M, Sugama S, Iwamaru Y, Kitani H, Hashimoto M (2010). P2X7 receptor signaling pathway as a therapeutic target for neurodegenerative diseases. Arch Immunol Ther Exp.

[100] Woods LT, Camden JM, Batek JM, Petris MJ, Erb L, Weisman GA (2012). P2X7 receptor activation induces inflammatory responses in salivary gland epithelium. Am J Phys Cell Phys.

[101] Sanz JM, Falzoni S, Rizzo R, Cipollone F, Zuliani G, Di Virgilio F (2014). Possible protective role of the 489C>T P2X7R polymorphism in Alzheimer's disease. Exp Gerontol.

[102] Sanz JM, Chiozzi P, Ferrari D, Colaianna M, Idzko M, Falzoni S, Fellin R, Trabace L, Di Virgilio F (2009). Activation of microglia by amyloid {beta} requires P2X7 receptor expression. J Immunol.

[103] Ortega F, Perez-Sen R, Morente V, Delicado EG, Miras-Portugal MT (2010). P2X7, NMDA and BDNF receptors converge on GSK3 phosphorylation and cooperate to promote survival in cerebellar granule neurons. Cell Mol Life Sci.

[104] Diaz-Hernandez JI, Gomez-Villafuertes R, León-Otegui M, Hontecillas-Prieto L, Del Puerto A, Trejo JL, Lucas JJ, Garrido JJ, Gualix J, Miras-Portugal MT, Diaz-Hernandez M (2012). In vivo P2X7 inhibition reduces amyloid plaques in Alzheimer's disease through GSK3β and secretases. Neurobiol Aging.

[105] Ryu JK, McLarnon JG (2008). Block of purinergic P2X(7) receptor is neuroprotective in an animal model of Alzheimer's disease. Neuroreport.

[106] Delarasse C, Auger R, Gonnord P, Fontaine B, Kanellopoulos JM (2011). The purinergic receptor P2X7 triggers alpha-secretase-dependent processing of the amyloid precursor protein. J Biol Chem.

[107] Leon-Otegui M, Gomez-Villafuertes R, Diaz-Hernandez JI, Diaz-Hernandez M, Miras-Portugal MT, Gualix J (2011). Opposite effects of P2X7 and P2Y2 nucleotide receptors on α-secretase-dependent APP processing in neuro-2a cells. FEBS Lett.

[108] Ford AP (2012). In pursuit of P2X3 antagonists: novel therapeutics for chronic pain and afferent sensitization. Purinergic Signal.

[109] Burnstock G (2006). Purinergic signalling. Br J Pharmacol.

[110] Moore D, Chambers J, Waldvogel H, Faull R, Emson P (2000). Regional and cellular distribution of the P2Y(1) purinergic receptor in the human brain: striking neuronal localisation. J Comp Neurol.

[111] Moore D, Iritani S, Chambers J, Emson P (2000). Immunohistochemical localization of the P2Y1 purinergic receptor in Alzheimer's disease. Neuroreport.

[112] Delekate A, Füchtemeier M, Schumacher T, Ulbrich C, Foddis M, Petzold GC (2014). Metabotropic P2Y1 receptor signalling mediates astrocytic hyperactivity in vivo in an Alzheimer's disease mouse model. Nat Commun.

[113] Weisman GA, Wang M, Kong Q, Chorna NE, Neary JT, Sun GY, Gonzalez FA, Seye CI, Erb L (2005). Molecular determinants of P2Y2 nucleotide receptor function: implications for proliferative and inflammatory pathways in astrocytes. Mol Neurobiol.

[114] Ajit D, Woods LT, Camden JM, Thebeau CN, El-Sayed FG, Greeson GW, Erb L, Petris MJ, Miller DC, Sun GY, Weisman GA (2014). Loss of P2Y_2_ nucleotide receptors enhances early pathology in the TgCRND8 mouse model of Alzheimer’s disease. Mol Neurobiol.

[115] Kong Q, Peterson TS, Baker O, Stanley E, Camden J, Seye CI, Erb L, Simonyi A, Wood WG, Sun GY, Weisman GA (2009). Interleukin-1beta enhances nucleotide-induced and alpha-secretase-dependent amyloid precursor protein processing in rat primary cortical neurons via up-regulation of the P2Y(2) receptor. J Neurochem.

[116] Kim HJ, Ajit D, Peterson TS, Wang Y, Camden JM, Gibson Wood W, Sun GY, Erb L, Petris M, Weisman GA (2012). Nucleotides released from Aβ_1-42_ -treated microglial cells increase cell migration and Aβ_1-42_ uptake through P2Y_2_ receptor activation. J Neurochem.

[117] Li HQ, Chen C, Dou Y, Wu HJ, Liu YJ, Lou HF, Zhang JM, Li XM, Wang H, Duan S (2013). P2Y4 receptor-mediated pinocytosis contributes to amyloid beta-induced self-uptake by microglia. Mol Cell Biol.

[118] Kettenmann H, Hanisch UK, Noda M, Verkhratsky A (2011). Physiology of microglia. Physiol Rev.

[119] Krabbe G, Halle A, Matyash V, Rinnenthal JL, Eom GD, Bernhardt U, Miller KR, Prokop S, Kettenmann H, Heppner FL (2013). Functional impairment of microglia coincides with Beta-amyloid deposition in mice with Alzheimer-like pathology. PLoS One.

[120] Bolmont T, Haiss F, Eicke D, Radde R, Mathis CA, Klunk WE, Kohsaka S, Jucker M, Calhoun ME (2008). Dynamics of the microglial/amyloid interaction indicate a role in plaque maintenance. J Neurosci.

[121] Streit WJ (2002). Microglia as neuroprotective, immunocompetent cells of the CNS. Glia.

[122] Streit WJ (2005). Microglia and neuroprotection: implications for Alzheimer's disease. Brain Res Brain Res Rev.

[123] Butovsky O, Talpalar AE, Ben-Yaakov K, Schwartz M (2005). Activation of microglia by aggregated beta-amyloid or lipopolysaccharide impairs MHC-II expression and renders them cytotoxic whereas IFN-gamma and IL-4 render them protective. Mol Cell Neurosci.

[124] Griffin WS (2006). Inflammation and neurodegenerative diseases. Am J Clin Nutr.

[125] Linden J (2005). Adenosine in tissue protection and tissue regeneration. Mol Pharmacol.

[126] Abbrachio MP, Ceruti S (2007). P1 receptors and cytokine secretion. Purinergic Signal.

[127] Elliott MR, Chekeni FB, Trampont PC, Lazarowski ER, Kadl A, Walk SF, Park D, Woodson RI, Ostankovich M, Sharma P, Lysiak JJ, Harden TK, Leitinger N, Ravichandran KS (2009). Nucleotides released by apoptotic cells act as a find-me signal to promote phagocytic clearance. Nature.

[128] Filiou MD, Arefin AS, Moscato P, Graeber MB (2014). 'Neuroinflammation' differs categorically from inflammation: transcriptomes of Alzheimer's disease, Parkinson's disease, schizophrenia and inflammatory diseases compared. Neurogenetics.

[129] Streit WJ, Xue QS, Tischer J, Bechmann I (2014). Microglial pathology. Acta Neuropathol Commun.

[130] Sugama S, Takenouchi T, Cho BP, Joh TH, Hashimoto M, Kitani H (2009). Possible roles of microglial cells for neurotoxicity in clinical neurodegenerative diseases and experimental animal models. Inflamm Allergy Drug Targets.

[131] Giaume C, Koulakoff A, Roux L, Holcman D, Rouach N (2010). Astroglial networks: a step further in neuroglial and gliovascular interactions. Nat Rev Neurosci.

[132] Marshak DR, Pesce SA, Stanley LC, Griffin WS (1992). Increased S100 beta neurotrophic activity in Alzheimer's disease temporal lobe. Neurobiol Aging.

[133] Pannasch U, Vargova L, Reingruber J, Ezan P, Holcman D, Giaume C, Sykova E, Rouach N (2011). Astroglial networks scale synaptic activity and plasticity. Proc Natl Acad Sci U S A.

[134] Franke H, Krügel U, Grosche J, Heine C, Härtig W, Allgaier C, Illes P (2004). P2Y receptor expression on astrocytes in the nucleus accumbens of rats. Neuroscience.

